# Temperature dependence of protein-water interactions in a gated yeast aquaporin

**DOI:** 10.1038/s41598-017-04180-z

**Published:** 2017-06-21

**Authors:** Camilo Aponte-Santamaría, Gerhard Fischer, Petra Båth, Richard Neutze, Bert L. de Groot

**Affiliations:** 1 Molecular Biomechanics Group, Heidelberg Institute for Theoretical Studies, Heidelberg, Germany; 2 Interdisciplinary Center for Scientific Computing (IWR), Heidelberg University, Heidelberg, Germany; 3 Department of Biochemistry, University of Cambridge, Cambridge, United Kingdom; 4 Department of Chemistry & Molecular Biology, University of Gothenburg, Gothenburg, Sweden; 5 Computational Biomolecular Dynamics Group, Max Planck Institute for Biophysical Chemistry, Göttingen, Germany; 6 Present Address: Max Planck Tandem Group in Computational Biophysics, University of Los Andes, Bogotá, Colombia

## Abstract

Regulation of aquaporins is a key process of living organisms to counteract sudden osmotic changes. Aqy1, which is a water transporting aquaporin of the yeast *Pichia pastoris*, is suggested to be gated by chemo-mechanical stimuli as a protective regulatory-response against rapid freezing. Here, we tested the influence of temperature by determining the X-ray structure of Aqy1 at room temperature (RT) at 1.3 Å resolution, and by exploring the structural dynamics of Aqy1 during freezing through molecular dynamics simulations. At ambient temperature and in a lipid bilayer, Aqy1 adopts a closed conformation that is globally better described by the RT than by the low-temperature (LT) crystal structure. Locally, for the blocking-residue Tyr31 and the water molecules inside the pore, both LT and RT data sets are consistent with the positions observed in the simulations at room-temperature. Moreover, as the temperature was lowered, Tyr31 adopted a conformation that more effectively blocked the channel, and its motion was accompanied by a temperature-driven rearrangement of the water molecules inside the channel. We therefore speculate that temperature drives Aqy1 from a loosely- to a tightly-blocked state. This analysis provides high-resolution structural evidence of the influence of temperature on membrane-transport channels.

## Introduction

Aquaporins are specialized transmembrane proteins which facilitate the transport of water across biological membranes and thereby maintain water homeostasis^1^. In addition to pure water transporters, the aquaporin family also includes closely related transporters of other small polar molecules, most frequently glycerol or larger sugar alcohols. Aquaporins are found in all kingdoms of life and fulfill a surprising variety of functions in the human body^2^, plants^3^, protozoa^4^, and microorganisms^5^.

Aquaporins are regulated on many different levels. Gradual changes of membrane water permeability can be controlled by the expression of aquaporins, whereas more rapid responses to external stresses are achieved by regulation through trafficking^6^. An even more rapid change in water transport activity can be achieved by gating^7,8^ whereby the water conducting pore of a gated aquaporin can be opened or closed through mechanical means, analogous to the opening and closing of a bottle with a hinged cap^9^. Aquaporin gating may be triggered by diverse types of external stimuli, including phosphorylation^10,11^ and pH^10,12,13^. Voltage^14^ and membrane-mediated mechanical stress^11^ have also been suggested as external factors inducing aquaporin gating.

Yeast aquaglyceroporins are believed to assist with the uptake of nutrients^8^ as well as buffering osmotic shock by transporting glycerol across the membrane^15^. Under more extreme conditions such as flash freezing (as may occur when rain falls upon frozen ground or as animals expel microorganisms in sub-zero temperatures) or during high osmotic shock (as when ripe fruit bursts or fresh-water raindrops land) aquaporins have been suggested to provide a protective function. In these circumstances aquaporins may provide a rapid outlet for water that prevents cells from bursting and thereby conveying a survival advantage to the host^11,16,17^.

*Pichia pastoris*, which is a methylotrophic yeast first discovered in tree bark but which is now better known as a protein overproduction host^18^, contains a water transporting aquaporin (called Aqy1) in its genome. The crystal structure of Aqy1 revealed that this aquaporin is gated by a tyrosine residue, Tyr31, located at the N-terminus, which enters the water channel from the cytoplasm and closes the pore to the passage of water^11^. Functional and mutational studies showed that Aqy1 could be regulated *in vivo* by the phosphorylation of Ser107 and molecular dynamics (MD) simulations also indicated that Aqy1 may be mechano-regulated by membrane-transmitted mechanical stress^11^. Furthermore, the survival of *Pichia pastoris* following multiple freeze/thaw cycles was compromised when Aqy1 was deleted^11^. Since the freezing of yeast cells in liquid nitrogen is extremely rapid, we hypothesized that Aqy1 might also be gated by mechano-stress during freezing.

In this work we sought to investigate the idea that Aqy1 may also be controlled by temperature during freezing, by comparing the crystallographic structure of Aqy1 recorded at room temperature (RT structure) with that previously recorded at 100 K (LT structure) and by performing MD simulations of Aqy1 at varying temperatures. Our data show that upon freezing, Tyr31 becomes more deeply buried within the cytoplasmic entrance into the aquaporin pore, suggesting that the Aqy1 water transport channel is loosely-closed at room temperature while tightly-closed when it is frozen. Only a small number of medium resolution membrane protein structures have been solved using classical crystallography methods at room temperature^19–21^, although this set is growing due to the advent of serial femtosecond crystallography at an X-ray free electron laser, which is a room-temperature method^22–27^. Our work, nevertheless, provides a high-resolution structural glimpse of the influence of temperature on membrane transport channels and on functional water-protein interactions.

## Results

### Room temperature data collection to 1.3 Å and structural refinement

**Figure 1:**
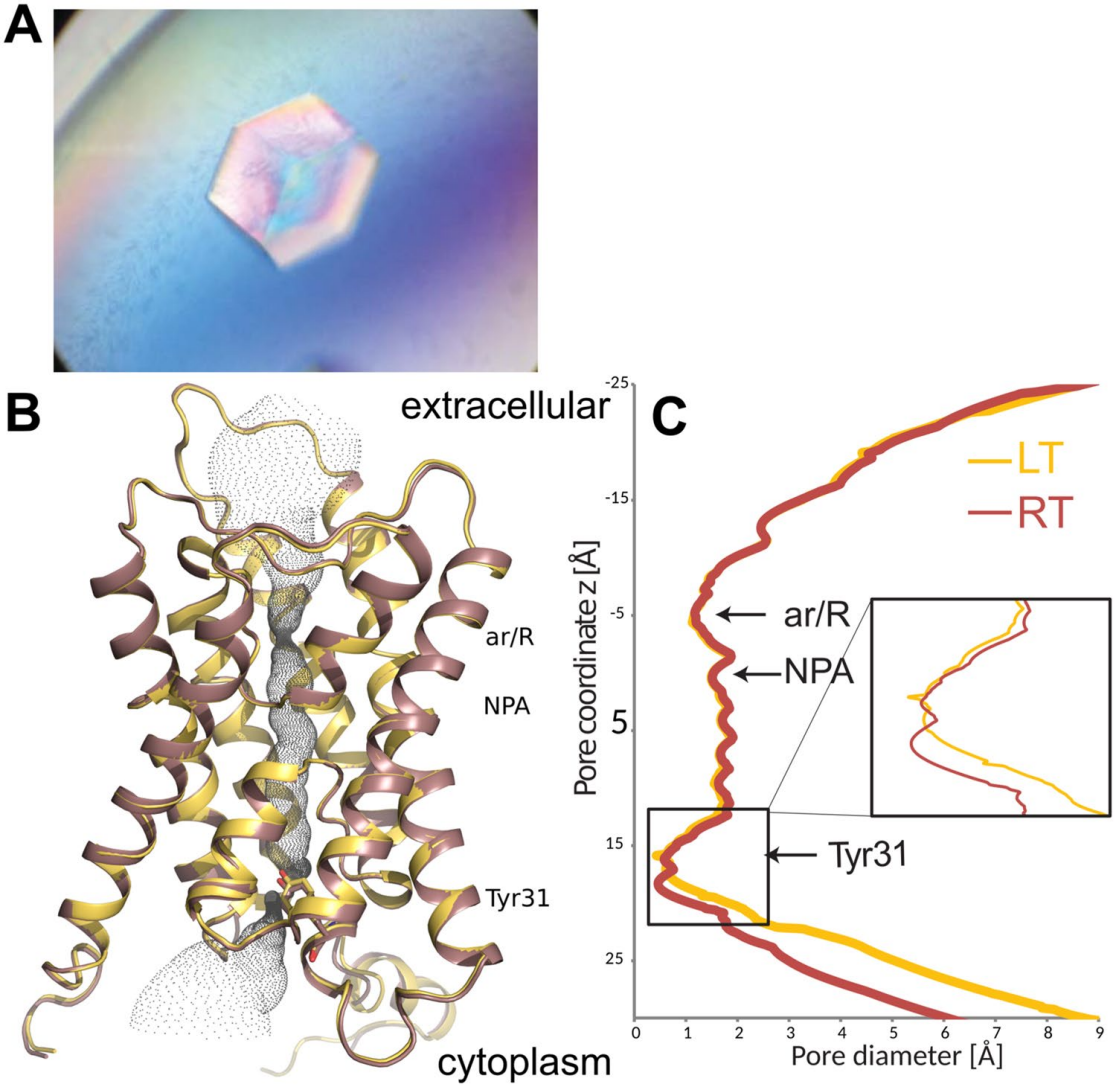
X-ray structure of Aqy1 at room temperature. (**A**) Crystal of *P*. *pastoris* aquaporin (**B**) Overlay of the X-ray structures of Aqy1 at 100 K (LT: PDB id. 3ZOJ, yellow) and at room temperature (RT: PDB id. 5BN2, red). The aquaporin signature NPA region and the aromatic/arginine selectivity filter (ar/R) inside the channel are indicated. The water pore outline is shown in grey. At the cytoplasmic side the channel is blocked by Tyr31. (**C**) Calculated pore diameter versus the z coordinate. The origin of the vertical coordinate is aligned to the NPA region.

Damaging effects of X-ray-induced radiation lead to loss of diffraction quality approximately two orders of magnitude faster at room temperature than at 100 K^28^. In order to overcome this limitation we developed new crystallization conditions that yielded significantly larger crystals (increased from 0.1 × 0.1 × 0.1 mm^3^ to 0.8 × 0.6 × 0.4 mm^3^, Fig. 1A) that grew at 20 °C. This circumvented problems encountered with crystals previously grown at 4 °C which cracked when the temperature was raised^11^. These larger crystals were able to tolerate 120 seconds of X-ray exposure with a nominal flux of 3 × 10^8^ photons/s when using a beam size of 0.3 × 0.2 mm^2^ at beamline I911-5 at MaxLAB II. Although approximately 50% of data collected from several crystals suffered from merohedral twinning, the best data set was recorded from a single crystal to 1.3 Å resolution and showed no signs of radiation damage, a very low mosaic spread of 0.11° and no twinning (Table 1). After molecular replacement using our earlier 0.88 Å resolution LT structure of Aqy1 as a search model (PDB code 3ZOJ), which was recorded from a crystal grown using identical crystallization conditions^29^, and several rounds of refinement against the room temperature data, we obtained an overall R_work_ = 11.2% with R_free_ = 14.3%. Only 98 water molecules were assigned against room temperature data, whereas 221 water molecules could be located in the electron density map at low temperature (Table 1).

**Table 1:** Crystallographic data collection and refinement statisti﻿cs.

PDB accession code	Aqy1 at Ambient Temperature
	5BN2
**Data Collection**	
Space group	I4
Cell dimensions	
a, b, c [Å]	92.49, 92.49, 81.19
α, β, γ [°]	90, 90, 90
Resolution^*^ [Å]	34.49–1.30 (1.37–1.30)
R_merge_	0.059 (0.499)
R_sym_	0.070 (0.592)
I/σ(I)	11.7 (2.4)
Completeness	99.6 (100)
Redundancy	3.4 (3.3)
**Refinement**	
Resolution [Å]	24.49–1.30
No. of reflections used	79248
R_work_/R_free_	11.4/14.3
No. of atoms	
protein	1983
heterogen	19
solvent	98
Overall Wilson/average B-factor	12.2/17.5
R.m.s. deviations	
Bond lengths [Å]	0.022
Bond angles [°]	1.9
Cruickshanks DPI [Å]	0.0287
Crystallization condition:	26% PEG600, 0.1 M Tris (pH = 8.0), 0.2 M CaCl_2_, 20 °C
Data collection temperature	ambient temperature (~293 K)

*Values in parenthesis are given for the highest resolution shell.

### Global overview of the room temperature structure

As expected, the RT structure of Aqy1 is very similar to Aqy1 structures obtained at 100 K (overlaid in Fig. 1B). Aqy1 arranges as a tetramer and each protomer folds as six transmembrane helices plus two half-helices that are formed by its elongated loops B and E. Both half-helices insert into the membrane, but from opposite sides, and align to create a seventh pseudo-transmembrane helix. The aquaporins’ dual NPA signature motif is located at the ends of these half-helices at the center of the water conducting pore approximately halfway through the membrane. Very similar pore-profiles were observed for the RT and LT Aqy1 structures (Fig. 1C). Overall, LT and RT protomer structures align with a root mean square deviation (RMSD) of 0.34 Å for 255 Cα-atoms, with most changes found in the N-terminal region. This value is significantly larger than the RMSD of 0.08 Å between the two LT structures (PDB codes 2W2E and 3ZOJ)^11,29^ crystallized under different conditions and different resolution.

**Figure 2:**
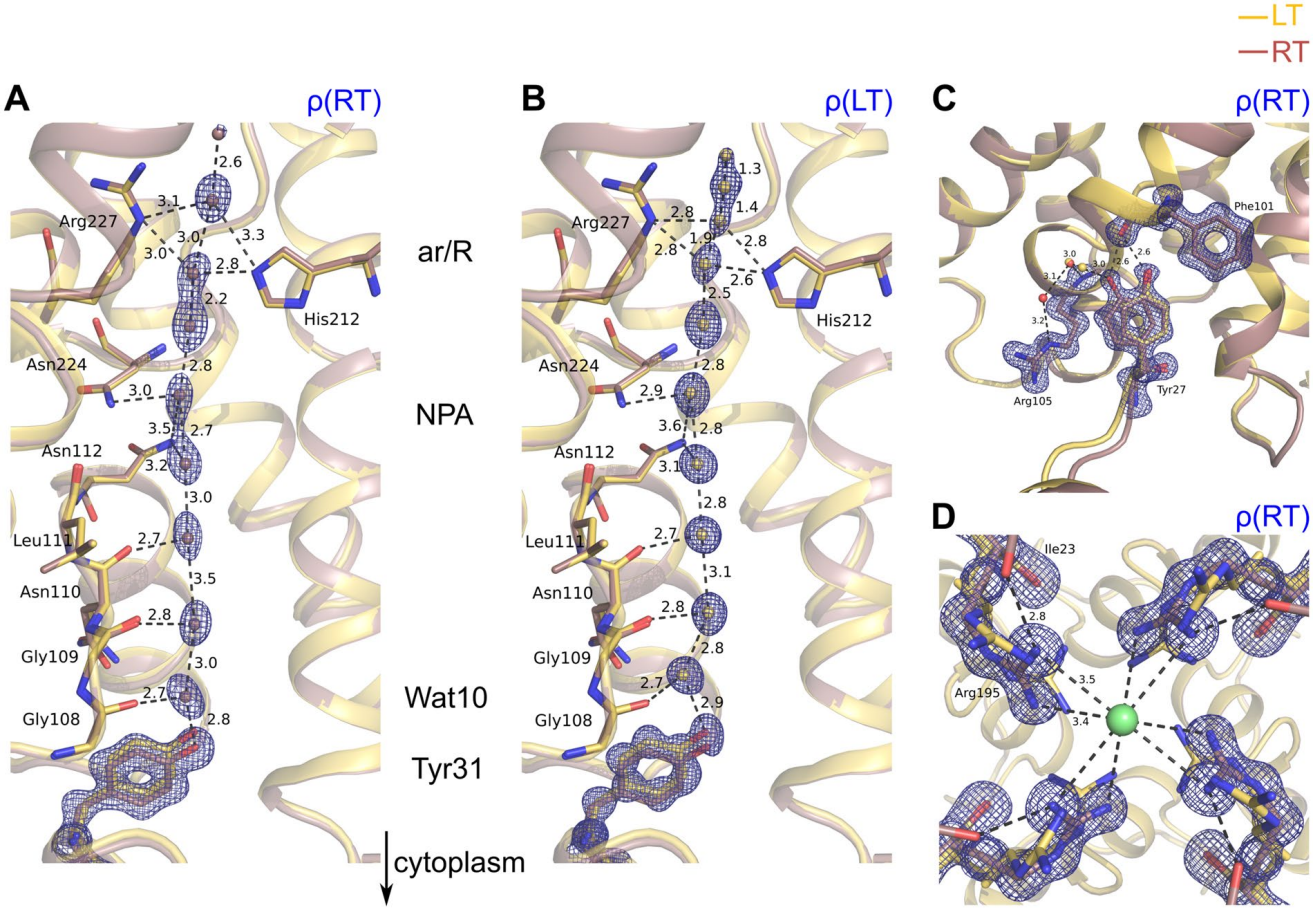
Electron density of Aqy1 determined by X-ray crystallography. (**A**,**B**) Electron density (blue mesh) of the water molecules inside the pore and of Tyr31 is shown for the RT-structure (RT, panel A) and compared to that of the LT-structure (LT, panel B). Structures have been aligned and are drawn as ribbon diagrams (RT: red and LT: yellow). Residues in direct contact with the water molecules are highlighted as sticks. The ar/R selectivity filter and the NPA constriction region are also indicated. Tyr31 is displaced further towards the cytoplasm at RT than at 100 K. The two water molecules closest to Tyr31 also move towards the cytoplasm at RT, with the water molecule closest to Tyr31 labelled Wat10. See quantitative difference in the positions of Tyr31 and the water molecules in Fig. 5A. (**C**) Tyr27, which plays a key role anchoring the N-terminus to the protein core, is observed to adopt two conformations at RT, yet only one of these conformations is occupied at 100 K. A new hydrogen bond network connecting Tyr27 to Arg105 via two water molecules is also visible at ambient temperature. (**D**) Four symmetry related Arg195-residues coordinating a single chloride ion in the central pore of the Aqy1 tetramer at room temperature. The chloride ion is located on the crystallographic 4-fold axis. In contrast, no density for the side chain of Arg195 is observed at LT (not shown). Arg195 also forms a hydrogen bond to Ile23 of the N-terminus in all protomers. In (**C** and **D**), the RT-electron density map is shown (blue mesh), with the structure model shown in red (RT) and yellow (LT). Interatomic distances in all panels are highlighted with the dashed lines and given in Å. All 2F_obs_-F_calc_ maps shown are contoured at 1.5 σ.

At ambient temperature, electron density is visible for several water molecules within the water transport pore (Fig. 2A). Apart from two waters molecules, which interact with Tyr31 and its nearest neighbor, all other water molecules within the channel have almost identical positions as observed in the low temperature Aqy1 structure (compare Fig. 2A with Fig. 2B). Moreover, unambiguous electron density is visible for all residues believed to play key roles in the mechanisms of solute permeation and proton exclusion by aquaporins, such as the asparagines of the dual NPA signature motif or the arginine of the conserved aromatic/arginine (ar/R) filter (Fig. 2A,B). This is an important finding since all models for the structural mechanism of permeation and proton exclusion by aquaporins have relied upon the atomic details of aquaporin crystal structures determined at low temperature. Had systematic deviations in the positions of key residues or water molecules emerged upon freezing, this would have impinged upon our understanding of proton and ion exclusion by these water selective channels. Interestingly, Tyr27, a residue anchoring the N-terminus onto the basic aquaporin scaffold, adopts one well defined conformation at 100 K, while it shows two conformations at ambient temperature (Fig. 2C). In addition to the dual Tyr27 conformation, the four Arg195 residues coordinate a chloride ion in the central pore of the Aqy1 tetramer at room temperature (Fig. 2D), but are not visible at low temperature.

### X-ray structural differences in light of the gating conformational changes

**Figure 3:**
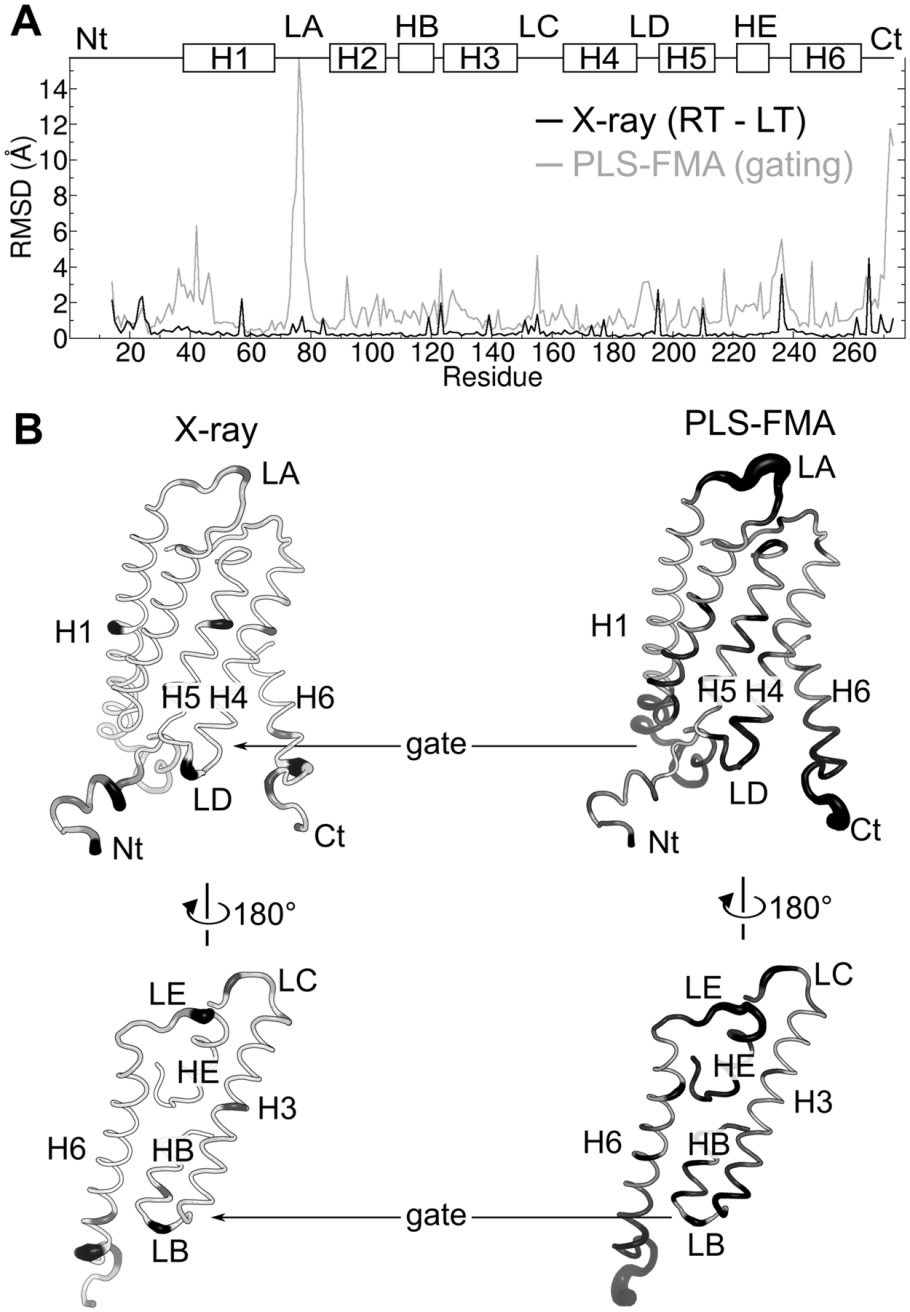
Structural differences between X-ray structures compared to gating motions of Aqy1. (**A**) Root mean square deviation (RMSD) as a function of the residue index is displayed. For the X-ray data, the RMSD quantifies the separation between the LT and RT structures (X-ray, black line). For the gating motion, which is induced by phosphorylation or membrane-mediated mechanical stress, the RMSD reflects the extent of the conformational changes during the opening motion, which was recovered by partial-least-square functional-mode-analysis (PLS-FMA, gray line). The secondary structure of the protein is displayed at the top, indicating helices (H1 to H6), half-helices (HB and HE), loops (LA to LD), and termini (Nt and Ct). See details of the RMSD calculations in the methods section. (**B**) Aqy1 monomer in cartoon-tube representation with the RMSD for each amino-acid encoded in the color and the size of the tube, ranging from RMSD = 0 (white and small) to RMSD > 2 Å (black and big). RMSD between the X-ray structures is shown at the left and RMSD associated to the gating transitions as revealed by PLS-FMA at the right. Two orientations are displayed for each case (not all secondary-structure elements are shown for clarity).

**Figure 4:**
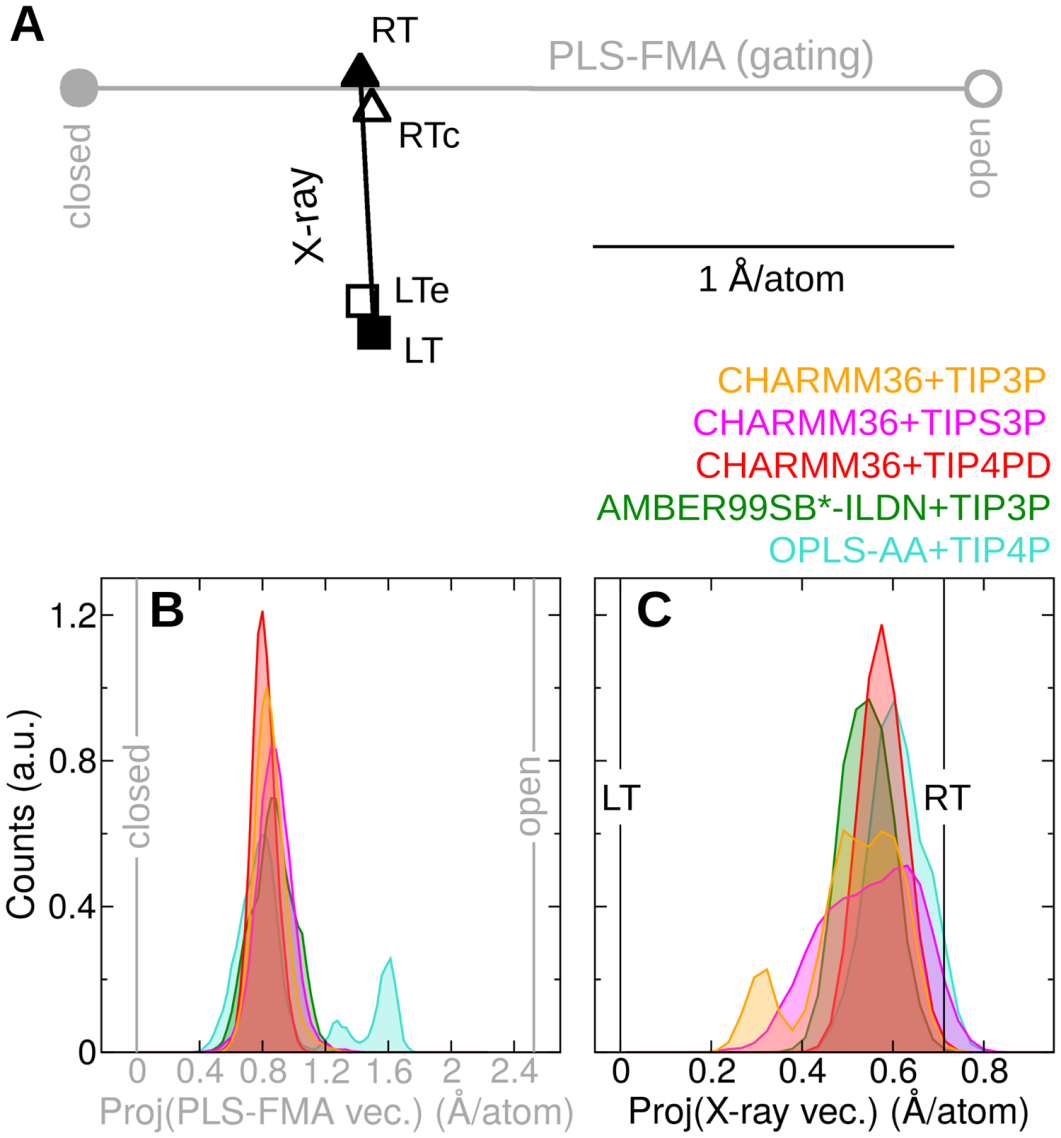
Conformational ensemble of Aqy1 at room temperature recovered from MD simulations. (**A**) Two-dimensional (2D) space constituted by vector associated to gating obtained by PLS-FMA (gray) and the vector connecting the X-ray structures (black). The PLS-FMA vector connects the extreme closed (filled circle) and open (empty circle) conformations. The X-ray vector connects the RT (filled triangle) with the LT (filled square) structures. These two vectors were found almost orthogonal (forming an angle of 86°). Projections of scaled conformations by a factor equal to the change in the crystallographic cell size during freezing are also displayed. Conformation at room temperature was compressed (RTc, empty triangle) while at low temperature was expanded (LTe, empty square). The horizontal black line represent a collective motion with each atom moving in average 1 Å. (**B**,**C**) Histograms of the projections of MD trajectories at room temperature along the PLS-FMA vector (**B**) and the X-ray vector (**C**) is presented for the five-used force-fields.

We next compared the structural variations between the two X-ray structures with the previously observed gating motions driven by phosphorylation or membrane-mediated mechanical stimulation^11^. Closer analysis of the gating transitions with partial least square functional mode analysis (PLS-FMA) revealed that collective opening motions spread over almost all secondary structure elements of the protein. During opening, large conformational variations expand beyond the gate of the pore, yielding RMSD larger than 2 Å, in all helices H1 to H6, the N and C termini, loops LA to LE and half-helices HB and HE (gray line in Fig. 3A and right cartoon in Fig. 3B). In contrast, the two X-ray structures are very similar, with RMSD between them smaller than 1 Å for most amino-acids (black line in Fig. 3A and left cartoon in Fig. 3B). Only few amino-acids display larger positional deviations, and such deviations were not found to be correlated with the fluctuations observed during gating (Fig. 3A,B). In fact, the collective vector connecting the X-ray structures was found to be almost perpendicular with the PLS-FMA collective vector associated to the gating transition (forming an angle of 86°, Fig. 4A).

Projections on the 2D-space constituted by the vector associated to the gating motion (PLS-FMA) and the vector connecting the X-ray structures allowed for further quantitative analysis. The X-ray structures consistently fall in the area corresponding to the closed state (filled symbols located more towards the left side of the 2D-space in Fig. 4A). Furthermore, the gating transition follows a path closer to the RT structure than to the LT structure (horizontal gray line crossing from left to right through the black triangle in Fig. 4A).

Equilibrium MD simulations using five different force-fields (see methods) confirmed the overall stability of the RT structure, with RMSDs from the simulations deviating not more than 2 Å from the initial conformation taken from the X-ray structure (Fig. S1A). Furthermore, qualitative good agreement was observed between the root mean square fluctuations (RMSFs) from simulations and the measured B-factors (Fig. S1B).

In addition, equilibrium MD simulations of Aqy1 embedded in a lipid bilayer corroborated that at room temperature this protein preferentially explores a conformational space near the (non-conducting) closed conformation as opposed to the (fully-conducting) open state seen during gating (Fig. 4B). More importantly, Aqy1 samples conformations closer to that of the X-ray RT structure (Fig. 4C). The same behavior was observed for the five different force fields, with one sporadic partial-opening event for OPLS-AA + TIP4P. The proximity of the conformational ensemble to the RT conformation is also independent on whether the starting conformation of the protein was taken from the RT or the LT structure (Fig. S2). Hence, our simulations support the conclusion that the crystallographic X-ray RT structure better represents the global closed state of Aqy1–under physiological conditions surrounded by a lipid bilayer and at ambient temperature.

Flash freezing the crystal shrank the crystallographic a and b axes (of equal length in space group I4) by 1.73 Å, from 92.49 Å to 90.76 Å. A similar reduction (1.47 Å) has been observed analyzing three and four additional data sets for room- and low temperature crystals, respectively. We checked the effect of this reduction by scaling all atomic positions by a factor equal to the change in cell size. The scaling brought the projections closer along the X-ray vector (compare filled with empty symbols in Fig. 4A). This indicates that shrinkage of the crystallographic cell is partially (but not fully) correlated with the differences between X-ray structures.

In summary, our comparison revealed that conformational changes between RT and LT structures are almost orthogonal to the conformational transitions observed during gating. The subtle variations between the X-ray structures cannot be entirely explained by changes of the crystallographic unit cell. Alternatively, they may be associated with temperature-sensitivity of Aqy1. This hypothesis is explored in detail in the following sections.

### Movement of the gating residue Tyr31 upon freezing

**Figure 5:**
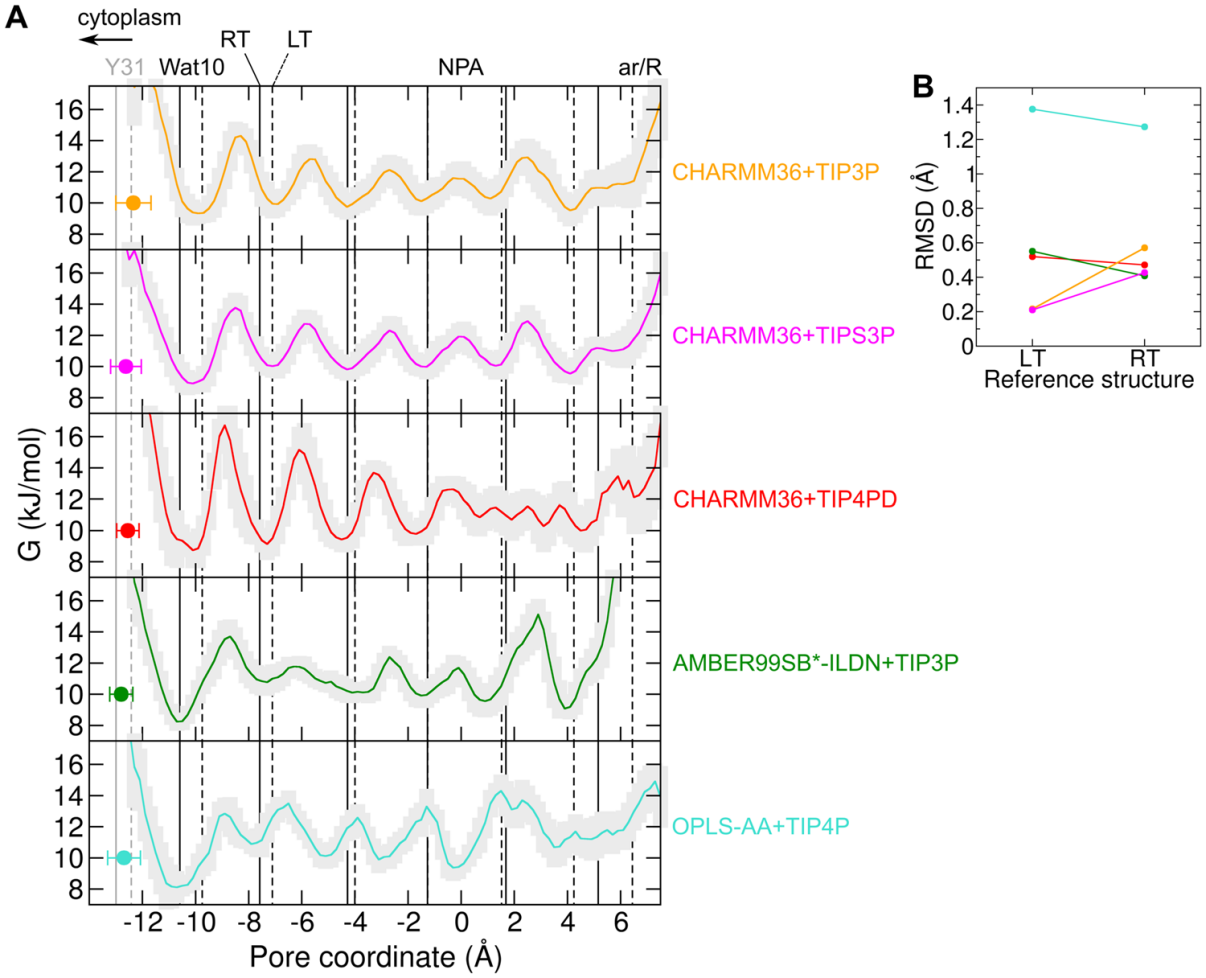
Positions of the gating residue Tyr31 and the water molecules inside the pore. (**A**) Potential of mean force (PMF) for the water permeation along the pore coordinate extracted from equilibrium MD simulations for the indicated force-fields (average: colored line, uncertainty: light gray shadow). The PMF minima indicate the most preferred positions of the water molecules throughout the simulations. Vertical lines represent the crystallographic positions in the RT structure (solid lines) and in the LT structure (dashed lines). Crystallographic positions of water molecules (including Wat10) are displayed in black and crystallographic position of the Tyr31 OH atom in gray. Tyr31 was found displaced towards the cytoplasm by about 0.5 Å at room temperature compared to low temperature. The two water molecules nearest Tyr31 also displaced towards the cytoplasm at room-temperature by about 0.5 and 0.8 Å, respectively. The latter displacement corresponds to Wat10. Average position ( ± standard deviation) along the pore coordinate of the Tyr31 OH atom recovered from the simulations is presented for comparison (colored dots). (**B**) Root mean square deviation (RMSD) of the PMF minima (most-favored water positions) with respect to the RT and LT crystallographic water positions is presented (same color-coding as in **A**).

Despite these overall similarities, local differences emerge between the high-resolution LT and RT structures of Aqy1. The residue occluding the pore, Tyr31, is displaced by 0.5 Å towards the cytoplasmic side in the RT structure compared to the LT structure (Figs 2A and 5A). This displacement causes an expansion of the column of waters inside the transport channel. When moving from the EC to the CP side of the channel, the water molecule positions in the RT structure only gradually deviate from those in the LT structure, with almost zero positional difference for the water located near Asn112 to as much as 0.8 Å displacement for the water molecule (Wat10) forming a direct hydrogen bond with Tyr31 (Figs 2A and 5A).

The observed movement of Tyr31 is not an isolated movement of one residue’s side-chain upon freezing, but rather it is correlated with a movement of the entire N-terminus, which is displaced by up to 2 Å in RMSD (Fig. 3A,B). Although the hydrogen bond network linking Tyr31 to Gly108 and Gly109 (Fig. 2A,B) and the pore-profiles remain almost intact (Fig. 1C), we may speculate that Tyr31 is more firmly locked in place at 100 K than at ambient temperature.

### Tyr31 and water-molecule localization inside Aqy1 from room temperature MD simulations

**Figure 6:**
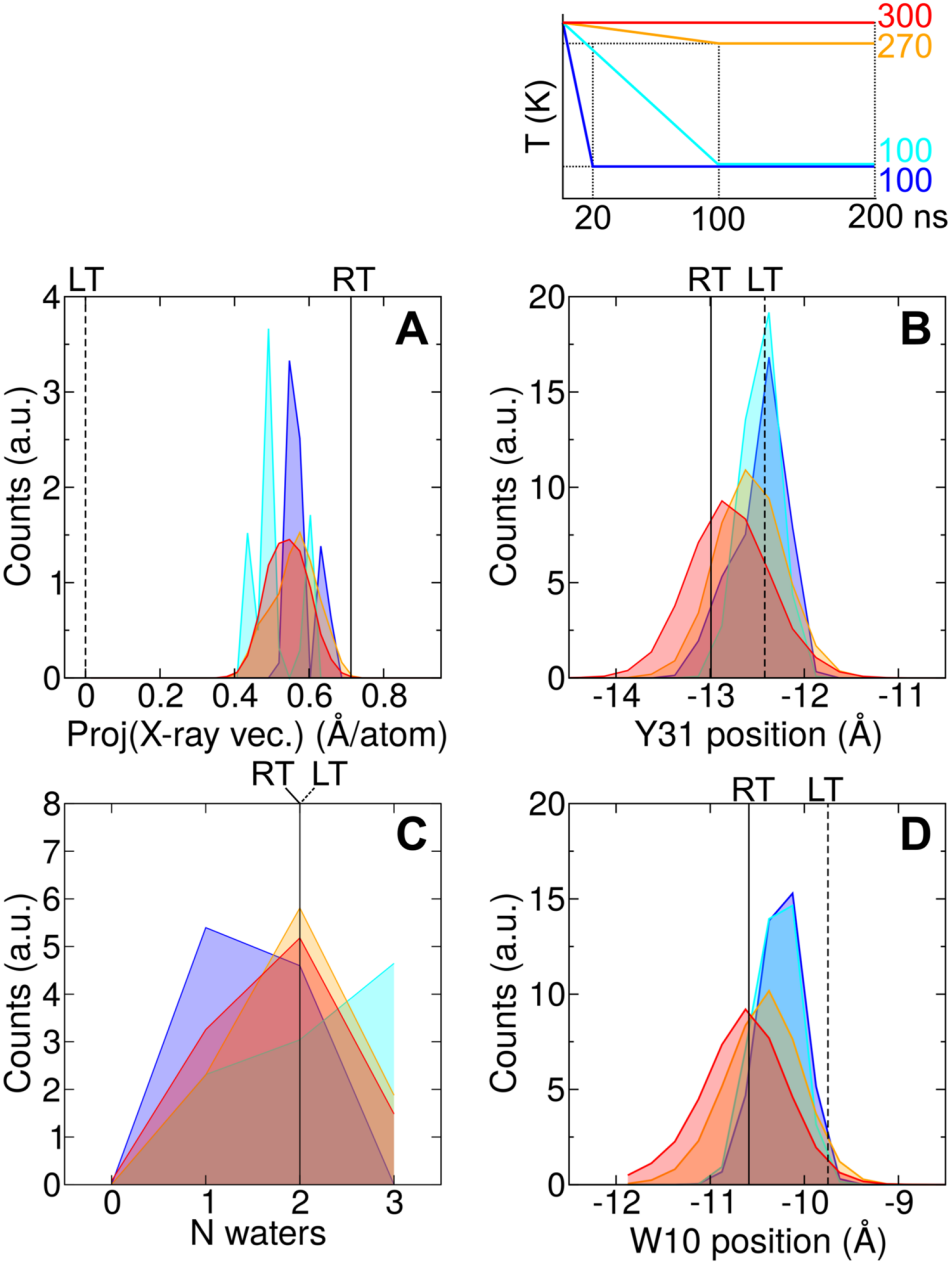
Aqy1 freezing simulations. Histograms of distinct quantities extracted from freezing MD simulations of Aqy1 are displayed. (**A**) Projection along the X-ray vector connecting the LT and RT structures. (**B**) Position of the Tyr31 OH atom along the pore coordinate with respect to the NPA region. (**C**) Number of waters coordinated by the asparagine residues Asn112 and Asn224 in the NPA region. (**D**) Position of the water molecule at crystallographic position Wat10 with respect to the NPA region. Vertical lines represent the values observed in the X-ray structures. Temperature was reduced from an initial value of 300 K to different values as indicated by the time-trace at the top. Histograms were computed during the last 80 ns of the simulations when the final temperature (different colors) had been reached. Histograms shown here correspond to the AMBER99SB*-ILDN-TIP3P force-field (see complete set of histograms for all force-fieds in Fig. S3).

During the MD simulations at room temperature, the Tyr31 OH atom was found to be localized in between the positions corresponding for this atom in the two X-ray structures (Fig. 5A). Because the positional fluctuations along the pore coordinate (0.4 to 0.7 Å) are comparable to the X-ray structural changes (0.5 Å), both the RT and the LT X-ray absolute positions are equally compatible with the simulated absolute position of Tyr31 OH at room temperature. Nevertheless, the relative displacement of this atom towards the NPA region upon reduction of the temperature has been consistently observed for all five force-fields (as discussed in detail in the next section and shown in Figs 6–S3).

Calculation of the potential of mean force (PMF) from the MD simulations revealed the most-favored positions of water molecules inside the channel at room temperature (PMF minima in Fig. 5A). For the crystallographic position Wat10, distances between PMF minima and X-ray positions remain below 1.0 Å, with a small bias (comparable to the resolution of the PMF minima of 0.4 Å) towards the LT position for the CHARMM36 simulations (independent of the water model) and towards the RT position for the AMBER99SB*-ILDN-TIP3P and OPLS-AA + TIP4P simulations. For the entire water column in the region between Tyr31 and the NPA motifs, water molecules accommodate at a maximum distance of 0.6 Å from the crystallographic positions, as revealed by the calculation of the RMSD between the PMF minima positions and the crystallographic positions (Fig. 5B). Furthermore, the difference in RMSD for the two reference structures (LT and RT) appear to be minimal (smaller than the resolution of the PMF minima of 0.4 Å). These two observations hold true for all force-fields except OPLS-AA + TIP4P for which the RMSD is larger than 1.2 Å. The large deviation for OPLS-AA + TIP4P could be attributed to the poor recapitulation of the water localization at the NPA region: one single water molecule –instead of two as observed with the other four force-fields and in the X-ray structures– is coordinated by both Asn112 and Asn224. Taken together, our PMF calculation show that both sets of crystallographic water positions (LT and RT) are consistent with the simulated positions of the water molecules inside the pore of Aqy1 when the protein is embedded in a lipidic environment at room temperature, with deviations between X-ray and simulation data sets in the sub-Angstrom regime.

### Freezing MD simulations

We also investigated the effect of reducing the temperature on the dynamics of Aqy1 and the water molecules inside its water transport pores by performing freezing MD simulations (Figs 6 and S3). Reduction of the temperature from 300 K to either 270 K or 100 K, during 100 ns or 20 ns long simulations, did not cause a substantial global conformational change of the protein (see projections of trajectories near the projection of the RT structure for all different ending temperatures in Figs 6A and S3A). This suggests that global conformational changes driven by temperature occur on time-scales longer than tens of nanoseconds. The gradual translation of Tyr31 was not clearly visible during the freezing process, presumably due to slow converging dynamics at low temperatures. Accordingly, Tyr31 could only be captured at an intermediate position, after reducing the temperature to 270 K, in only one out of the five simulations (see histogram for 270 K in between the histograms for 300 K and 100 K for AMBER99SB*-ILDN-TIP3P in Figs 6B and S3B). Nevertheless, freezing to 100 K induced Tyr31 to populate exclusively deeper positions inside the channel, in concordance with the difference in positions observed for this residue in the X-ray structures (compare histograms for 100 K with those for 300 K and 270 K in Figs 6B and S3B). Therefore, our simulations qualitatively support the notion of temperature controlling the extent to which Tyr31 is buried into the channel.

We quantified the number of water molecules coordinated at the NPA region by the asparagine residues Asn112 and Asn224 (Figs 6C and S3C). This quantity displays large sensitivity to the used force-field. At high temperatures, AMBER99SB*-ILDN-TIP3P favors two water molecules coordinated by the asparagines, OPLS-AA + TIP4P prefers one, while CHARMM36-TIP4PD varies between one and two. At low temperature, the three force-fields predominantly show a higher probability for a coordination of two water molecules, with a non-negligible probability also for one or three coordinated waters molecules in the AMBER99SB*-ILDN-TIP3P simulations. The overall trend, either one or two water molecules coordinated by the NPA-asparagines at room temperature shifting towards two water molecules at low temperatures, is in agreement with what has been observed in the X-ray structures (compare vertical lines with peaks of distributions in Fig. 6C). The water molecule at the crystallographic position Wat10 was located further towards the cytoplasmic side in the simulations at high temperatures than in those at low temperatures (Fig. 6D) for all the five used force fields (Fig. S3D). Accordingly, its motion has been found to be correlated with the position of Tyr31 (compare Fig. 6B and D and Fig. S3B and D). In consequence, our simulations at different temperatures are consistent with a temperature-driven expanding and contracting water column inside Aqy1 together with the motion of Tyr31.

## Discussion

The number of protein structures solved at room temperature^19–21^ is growing due to the advent of serial femtosecond crystallography techniques^22–27^. Our work complements these studies by providing a high-resolution structural glimpse of the influence of temperature on membrane transport channels and on functional water-protein interactions.

Our simulation data support that Aqy1 preferentially adopts a global conformation similar to that observed in the X-ray RT structure when it is embedded in a lipidic environment, under equilibrium conditions, and at room temperature. Locally, both LT and RT crystallographic data sets are compatible with the positions of Tyr31 and the water molecules inside the pore, with minimal differences in the sub-Angstrom regime. In this equilibrium state, this protein is ready to be opened by chemo-mechanical stimulation, such as phosphorylation or membrane-mediated mechanical stress. Sporadic opening transitions may still occur in the absence of such stimuli. This is consistent with water transport assays of Aqy1 *in vivo*, indicating residual water transport activity for full length Aqy1, that is only a sixth of that observed for the fully open Y31A Aqy1 mutant and for the Aqy1ΔN36 construct (in which the first 36 residues of the N-terminus were removed)^11^. Global structural changes between RT and LT X-ray structures do not show a clear correlation with conformational motions during gating observed previously^11^, suggesting temperature-induced conformational changes to be orthogonal to phosphorylation- or mechanically-induced gating transitions of Aqy1.

Scaling of the X-ray structures by a factor equal to the cell size change upon flash freezing brought conformations closer, but not to a full overlap. This implies that shrinkage of the crystallographic cell only in part correlates with the observed global conformational changes. In consequence, other crystallographic factors must still be at play to amplify the effect of volume changes. Moreover, the fact that the collective vector connecting the X-ray structures is orthogonal to that associated to gating indicates that the mechanical perturbation necessary to open the channel is not attained by freezing the crystals. Within the crystals, each Aqy1 molecule is surrounded by detergent micelles. Detergent micelles keep the protein in solution during purification and crystallization but they only partially mimic the properties of a natural lipidic membrane. Thus while micelles successfully shield the hydrophobic part of the protein from water they do not account for the membrane tension exerted by a lipid bilayer *in vivo*, or *in silico* during the MD simulations.

One captured local motion during freezing seems to be of high relevance: the tyrosine residue blocking the channel at the cytoplasmic side, Tyr31, is found displaced by ~0.5 Å towards the cytoplasm at room temperature compared to its position at cryogenic temperature. Such a subtle change caused an expansion of half of the pore’s water column between Tyr31 and the NPA region. Slow converging protein dynamics in the freezing simulations may have prevented us from directly observing the gradual shift in position of Tyr31 by reducing the temperature. Because of that, we were only able to detect Tyr31 at an intermediate position after decreasing the temperature from 300 K to 270 K in one out of the five simulation data sets. Nevertheless, our simulations successfully captured both the relative displacements of Tyr31 and the further accommodation of the water column inside the channel, by reducing to a cryogenic temperature of 100 K. As a consequence, our simulation data suggest that the detected motion of Tyr31 may not be an artifact of the crystallization conditions but a response to changes in temperature. We may speculate that temperature drives Aqy1 from a loosely-blocked to a tightly-blocked state by controlling the degree by which Tyr31 is buried inside the channel.

Although structural biology has its roots in room-temperature data-collection, the popularity of ambient temperature methods has long been superseded by data collection at cryogenic temperatures. Here, by growing larger crystals which reduce the X-ray exposure per frame and thereby enhance the amount of data that can be collected before the influence of radiation damage becomes apparent, we could address questions concerning the physiological relevance of cryogenic low-temperature X-ray structures of aquaporins. Previous MD studies evaluated the impact of several crystallographic conditions such as the packing^30,31^, use of detergents^30^, and protein structural modifications^32^. Our work expands these studies by assessing the effect of another critical crystallographic factor, namely the temperature. The global structure of the protein at room temperature was highly similar to previous structures determined at low temperature, with differences only in the sub-Angstrom regime. This is an important confirmation, because all structural mechanisms proposed so far for Aqy1 are assumed to hold at ambient temperature but have been based on cryogenic-temperature structural data. The highly conserved structural-fold of aquaporins suggests that this observation may also be extended to the complete aquaporin family^8^. Nevertheless, localization of critical residues and water molecules may differ, as it is indeed the case for Tyr31 and the water molecules inside the pore of Aqy1. Thus, our results suggest to critically assess the potential effects of temperature, before interpreting cryogenic low-temperature X-ray structures as representatives of native conditions at ambient temperature. Ambient X-ray crystallography combined with freezing MD simulations offers an attractive approach to perform this assessment.

Water localization inside Aqy1 also turned out to be a critical test for MD simulation force-fields. In our case, it was necessary to employ five different force-fields to reach a consensus picture of the effects of temperature. Although the global dynamics of the protein displayed little sensitivity to the used force-field, the absolute position of the Tyr31 and the position of waters inside the channel were more susceptible. In particular at room temperature, the localization of the water molecule adjacent to Tyr31 and the number of water molecules coordinated by the asparagine residues at the NPA region were found to vary across different force-fields. However, the five force-fields consistently showed similar temperature-dependent relative positioning of Tyr31 and the water column reorganization. Their trends are comparable in relative terms with the X-ray positions. Our simulations also provide evidence that the TIP4P-D water model, initially designed to reproduce conformational ensembles of intrinsically disordered proteins, captures the dynamics of membrane proteins similarly well as conventional water models designed for this purpose (TIP3P, TIPS3P, and TIP4P). Nevertheless, TIP4P-D displayed a stronger water localization (evidenced in energetic barriers higher than the value of ~4 kJ/mol observed for the other force-fields shown in Fig. 5A). We think that a much stronger coordination of the water molecule adjacent to Tyr31 may be responsible for that. This is an interesting finding, as it specifically presents a distinct behavior of this water model influencing water-protein interactions in confined environments. Water localization inside aquaporins obeys a delicate balance between protein-water and water-water interactions, which are dynamically changing and competing^33^. Hence, the ability to correctly reproduce this balance, and thereby to faithfully predict the water localization, constitutes a critical and challenging check for validation and improvement of biomolecular MD-simulation force-fields. In the particular case of Aqy1, X-ray water molecules inside its pore are well localized and therefore this may constitute a good reference system to perform this check.

## Conclusion

We investigated the effect of temperature on the structure of Aqy1, by means of ambient-temperature X-ray crystallography and MD simulations. Although globally the structure at room temperature is almost identical to those previously determined at cryogenic low temperature, local sub-Angstrom temperature-dependent conformational changes were observed. In particular, the position of the tyrosine residue blocking the channel, Tyr31, was found to vary with temperature, causing a rearrangement of the water column inside the channel. Control by temperature of the extent by which this residue is buried inside the channel may be a plausible mechanism to drive the channel from a fully-locked to a loosely-locked state. It will be highly interesting to test this hypothesis, along with its physiological connection to chemo-mechanical gating, in future studies. Our results provide relevant high-resolution structural evidence of the influence of temperature on water-protein interactions in membrane protein channels. We suggest to critically assess sub-Angstrom structural variations induced by temperature variations, before interpreting atomic positions derived from cyogenic-temperature X-ray-crystallography.

## Methods

### Protein Production and Purification

A non-tagged version of Aqy1 was recombinantly overproduced in *P*. *pastoris* using 3 L bioreactors and MeOH induction as described previously^11^. Briefly, cells were lysed using X-press equipment in a frozen suspension (50 mM Tris, pH = 8.0, 5% glycerol). The non-membrane fraction was removed by centrifugation at 12000 g, 20 min, 4 °C. The membrane fraction was collected from the supernatant using ultacentrifugation (150000 g, 2 h, 4 °C), resuspended, washed in 20 mM NaOH solution and collected again using ultracentrifugation (150000 g, 2 h, 4 °C). Approximately 1 g of the membrane pellet was then solubilized in 50 mL of 5% n-octyl-β-D-glucopyranoside (β-OG, Anatrace), 20 mM Tris (pH = 8.0), 20 mM NaCl, 10% glycerol, 1 tablet of protease inhibitor cocktail (Complete EDTA-free, Roche Diagnostics) for 45 minutes at ambient temperature. Ion-exchange chromatography (6 mL ResourceQ column, GE Healthcare) with a 15 column volume NaCl gradient (0 mM to 500 mM) in 1% β-OG, 20 mM Tris (pH = 8.3) was used as primary purification step. Before crystallization, the sample buffer was exchanged to 1% β-OG, 10 mM HEPES (pH = 7.0), 100 mM NaCl using size exclusion chromatography (Superdex 200 16/60, GE Healthcare) and concentrated using a 50 kMWCO Vivaspin concentrator tube.

### Crystallization and Data Collection

Initial crystals were obtained at 4 °C^11^. These were optimized to grow at 20 °C in 26% PEG600, 100 mM Tris (pH = 8.0), 200 mM CaCl_2_ in a sitting drop crystallization setup^29^. Drops of 20 μL at a protein concentration of 5 mg/mL were mixed with 5 μL of precipitant solution within one day after purification. These crystallization plates were transported by car to MaxLab II, Sweden. Crystals were harvested from the trays using Litholoops (Mitegen) and sealed together with some well solution using MciroRT polymer capillaries (Mitegen). Diffraction data from these crystals was collected at MaxLab II, Sweden, beamline I-911-5, at a wavelength of 0.91 Å.

### Data Processing and Refinement

Data processing was performed using the CCP4 program suite^34^. The diffraction images were integrated to 1.3 Å using iMosflm 1.0.5^35^ and scaled using SCALA. The phase problem was solved using Molecular Replacement (PHASER)^36^ with the low temperature structure of Aqy1 (PDB code: 3ZOJ) as a model. This model was then iteratively refined using Coot^37^ and Refmac^38^. 97.9% of all residues are located in the favored and 1.3% in the allowed regions of the Ramachandran plot, with 2 residues (Asn221 and Asn224 of the characteristic NPA region) as outliers. Further statistics are shown in Table 1. RMSD calculations between structures were carried out with PyMol^39^. The effective pore diameter was calculated using HOLE^40^.

### Molecular dynamics simulations

Molecular dynamics (MD) simulations were carried out as described in our previous study^11^ by using the GROMACS software (4.5.3 and 5.1.1 versions)^41,42^. Five different force-fields were employed: AMBER99SB*-ILDN^43–45^ with the TIP3P^46^ water model; CHARMM36^47,48^ with TIP3P^46^, TIPS3P^47^, and TIP4PD^49^ water parameters, and OPLS-AA^50,51^ with the TIP4P^46^ water force-field. The simulated system consisted of the Aqy1 tetramer embedded in a fully solvated lipid bilayer of approximately 279 palmitoyloleoylphosphatidylethanolamine (POPE) lipids (289 palmitoyloleoylphosphatidylcholine, POPC, lipids for the case of AMBER99SB*-ILDN-TIP3P). Although in the latter case, we used a lipid with a different head-group, based on work from others^52–54^ and us^31,55^, we do not think it should severely influence the protein-water dynamics at the conduction pores (see Supplementary text S1). The bilayer was fully solvated with 21884 (AMBER99SB*-ILDN-TIP3P), 18945 (CHARMM36-TIP3P and CHARMM36-TIPS3P), 19816 (CHARMM36-TIP4PD), and 16513 (OPLS-AA-TIP4P) explicit water molecules. Crystallographic waters were also kept in the simulations. Sodium and chloride ions were added at a 150 mM concentration, and additional chloride ions to neutralize the charge of the protein. Initial coordinates of the protein atoms were taken from the X-ray structures of Aqy1 at room temperature (RT). Two additional simulations were carried out from the low temperature structure, either without refinement (PDB code 2W2E)^11^, referred here as LT_NR_, or after refinement (PDB code 3ZOJ)^29^. These two additional simulations were carried out with the OPLS-AA-TIP4 force-field. The tetramer was embedded in the bilayer using g_membed^56,57^. Lipid parameters were taken Berger *et al*.^58^ for the simulations OPLS-AA-TIP4P, modified according to Cordomí *et al*.^59,60^ to be used with AMBER99SB*-ILDN-TIP3P. CHARMM36 lipids^61^ were used for the simulations with CHARMM36 force-field (with the three water models). Default ion parameters were considered, except for AMBER99SB*-ILDN-TIP3P for which parameters were taken from Joung *et al*.^62^. Bond lengths involving hydrogen atoms were constrained in the protein by using LINCS^63^. In the case of OPLS-AA-TIP4P, all bonds^63^ as well as angles involving hydrogen atoms^64^ were constrained. Water molecules were constrained by using Settle^65^. Electrostatic interactions were treated by using the particle-mesh Ewald method^66,67^. Short-range dispersion and attractive interactions were considered by a Lennard-Jones potential. They were considered within a cutoff distance of 1.0 nm (1.2 nm for CHARMM36-TIP4PD). They were shifted to zero at the cutoff distance for AMBER99SB*-ILDN-TIP3P and CHARMM36-TIP4PD. Force derived from the Lennard-Jones potential was smoothed to zero between 1.0 nm and 1.2 nm for CHARMM36-TIP3P and CHARMM36-TIPS3P. Verlet Buffer particle-particle neighbor scheme^68^ was taken into account (except for the simulation with OPLS-AA-TIP4P, for which standard cut-off scheme was applied). Equations of motion were integrated at discrete time steps of 2 fs (4 for the OPLS-AA-TIP4P simulation). The temperature was maintained at 300 K by coupling the system to the v-rescale thermostat^69^ with coupling constant τ = 1 ps (τ = 0.5 ps for OPLS-AA-TIP4P). The pressure was kept constant at 1 bar by using a semi-isotropic Parrinello-Rahman barostat^70^ (coupling constant τ = 5 ps). The lipids and water molecules were equilibrated around the protein during 4 ns before the production runs, maintaining the protein’s heavy atoms and crystallographic water oxygens harmonically restrained (harmonic elastic constant k = 1000 kJmol^−1^nm^−2^). Position restraints were released and the dynamics of the protein tetramer was monitored during 200 ns (100 ns for OPLS-AA-TIP4P). Each monomer contained a water channel and constituted an independent water conducting unit, therefore each simulation resulted in a combined sampling of 800 ns (400 ns for OPLS-AA-TIP4P) of monomer dynamics. First 20% of each simulation was discarded and accounted as equilibration time.

Freezing simulations were carried out starting from the RT protein conformation (after equilibration of the solvent). The temperature was linearly decreased from an initial temperature of 300 K to a final temperature of either 100 K (during 20 or 100 ns) or 270 K (during 100 ns). After freezing, the temperature was maintained constant at the final temperature until 200 ns were reached (183 ns for a reduction to 100 K during 100 ns using the CHARMM36-TIPS3P force-field). Same simulation parameters were used as in the simulations at 300 K (except for the temperature change). Histograms were collected in the last 80 ns of the simulations, when the final temperature was already reached.

A collective vector, ***P***, associated to the closed to open gating transitions of Aqy1, was considered. It was computed by using partial least square functional mode analysis (PLS-FMA), as in the previous study^71^, but this time considering all atoms except hydrogens. This vector is termed as PLS-FMA throughout the text. Principal component analysis^72^, consisting on the calculation and diagonalization of the covariance matrix of the heavy-atom positions, was computed to obtain a second collective vector, ***X***, which connects the X-ray RT and LT structures. In the text this vector is named X-ray vector. The root mean square deviations (RMSD) was computed as a measure of the magnitude of the conformational change for each amino-acid along these collective vectors. Conformations extracted from the X-ray RT and LT structures, as well as extreme closed (C) and open (O) conformations separating the vector ***P***, were projected on both ***X*** and ***P*** vectors. This gave scalar projections *proj*(*T*;***V***), with *T* = LT, RT, C, or the O conformation, and ***V*** = ***X*** or the ***P*** vector. The angle α formed by ***X*** and ***P*** was determined from cosα = [*proj*(RT;***P***)–*proj*(LT;***P***)]/[*proj*(RT;***X***)–*proj*(LT;***X***)]. Conformations sampled during the MD simulations, *r*, were represented as points with coordinates (*r*_*x*_, *r*_*y*_) onto the 2-dimensional space formed by ***X*** and ***P***. To take into account the angle α between these vectors, such coordinates were given by (Fig. S4):

$${r}_{x}=proj(r;{\boldsymbol{P}})-proj({\rm{C}};{\boldsymbol{P}}),\,{\rm{and}}\,{r}_{y}=b-e.$$

Here, *b* = *c*sinα, where *c* = *proj*(*r*;***X***) − *proj*(LT;***X***), and *e* = *d*/tanα, where *d* = *proj*(*r*;***P***) − *proj*(*LT*;***P***) − *a*, with *a* = *c*cosα. Note that for the particular case of the conformation C, *r*_*x*_ = 0, while for the conformation LT, *r*_*y*_ = 0. Thus the C and LT conformations define the origin of the 2-dimensional projection space in the *x*- and *y*-axis, respectively.

The potential of mean force, *PMF*, associated to the permeation of water, was assessed using the equation^73^: *PMF*(*z*) = −*k*_B_ *T*ln < *n*(*z*)>, where *k*_B_ is the Boltzmann constant, *T* is the temperature, and <*n*(*z*)> is the time-averaged water density as a function of the pore coordinate *z*. Because the number of realizations to compute the water density was very large, the PMF estimation using this formula approximated very well with that computed using umbrella sampling^74^. Profiles presented in Fig. 5A were computed as the exponential average exp(−<*PMF*(*z*) > /*k*_B_ *T*) = (1/4)Σ_i=1_^4^exp(−<*PMF*_*i*_(*z*) > /*k*_B_ *T*), derived from 4 monomer profiles, *PMF*_*i*_(*z*), taken from each 300 K simulation. Accordingly, its uncertainty was estimated by propagating the error of the exponential average. A trapezoidal correction was applied to the potentials to account for the transversal effective area of the monomers^75^. Near the ar/R region the PMF displayed an increased barrier due to occasional flips of the residues lining the pore. This unclear effect, identified earlier for Aqy1^11^, was found uncorrelated to the water localization and energetic barriers at the opposite side of the channel (between the Tyr31 residue and the NPA region). The root mean square deviation (RMSD) of the PMF minima to the closest crystallographic water position was computed considering the five waters molecules spanning the region between Tyr31 and the NPA region. The RMSD was computed separately taking the either the RT or the LT as reference crystallographic positions. The occupancy of the NPA region was computed as the number of water molecules located inside a region spanning 0.4 nm of the pore aligned with the center of mass of the NPA motifs.

## Electronic supplementary material

Supplementary material
