# Author Correction: Temperature dependence of protein-water interactions in a gated yeast aquaporin

**DOI:** 10.1038/s41598-017-17040-7

**Published:** 2017-12-05

**Authors:** Camilo Aponte-Santamaría, Gerhard Fischer, Petra Båth, Richard Neutze, Bert L. de Groot

**Affiliations:** 1 Molecular Biomechanics Group, Heidelberg Institute for Theoretical Studies, Heidelberg, Germany; 2 Interdisciplinary Center for Scientific Computing (IWR), Heidelberg University, Heidelberg, Germany; 3 Department of Biochemistry, University of Cambridge, Cambridge, United Kingdom; 4 Department of Chemistry & Molecular Biology, University of Gothenburg, Gothenburg, Sweden; 5 Computational Biomolecular Dynamics Group, Max Planck Institute for Biophysical Chemistry, Göttingen, Germany; 6 Present Address: Max Planck Tandem Group in Computational Biophysics, University of Los Andes, Bogotá, Colombia

*Scientific Reports* **7**:4016; doi:10.1038/s41598-017-04180-z; Article published online 21 June 2017.

The original version of this Article contained an error in Affiliation 6, which was incorrectly given as:

‘Planck Tandem Group in Computational Biophysics, University of Los Andes, Bogotá, Colombia’.

The correct affiliation is listed below:

‘Max Planck Tandem Group in Computational Biophysics, University of Los Andes, Bogotá, Colombia’.

This error has now been corrected in the HTML and PDF versions of the Article.

