# Supplementary material for: Temperature dependence of protein-water interactions in a gated yeast aquaporin
Source: Sci Rep. 2017 Jun 21;7:4016. doi: 10.1038/s41598-017-04180-z (PMC5479825; doi:10.1038/s41598-017-04180-z)
Supplement: Supplementary file 1 — Supplementary material [file 41598_2017_4180_MOESM1_ESM.pdf]

SUPPLEMENTARY MATERIAL FOR:

Temperature dependence of protein-water interactions in a gated yeast aquaporin

Camilo Aponte-Santamaría, Gerhard Fischer, Petra Båth, Richard Neutze, and Bert L. de Groot

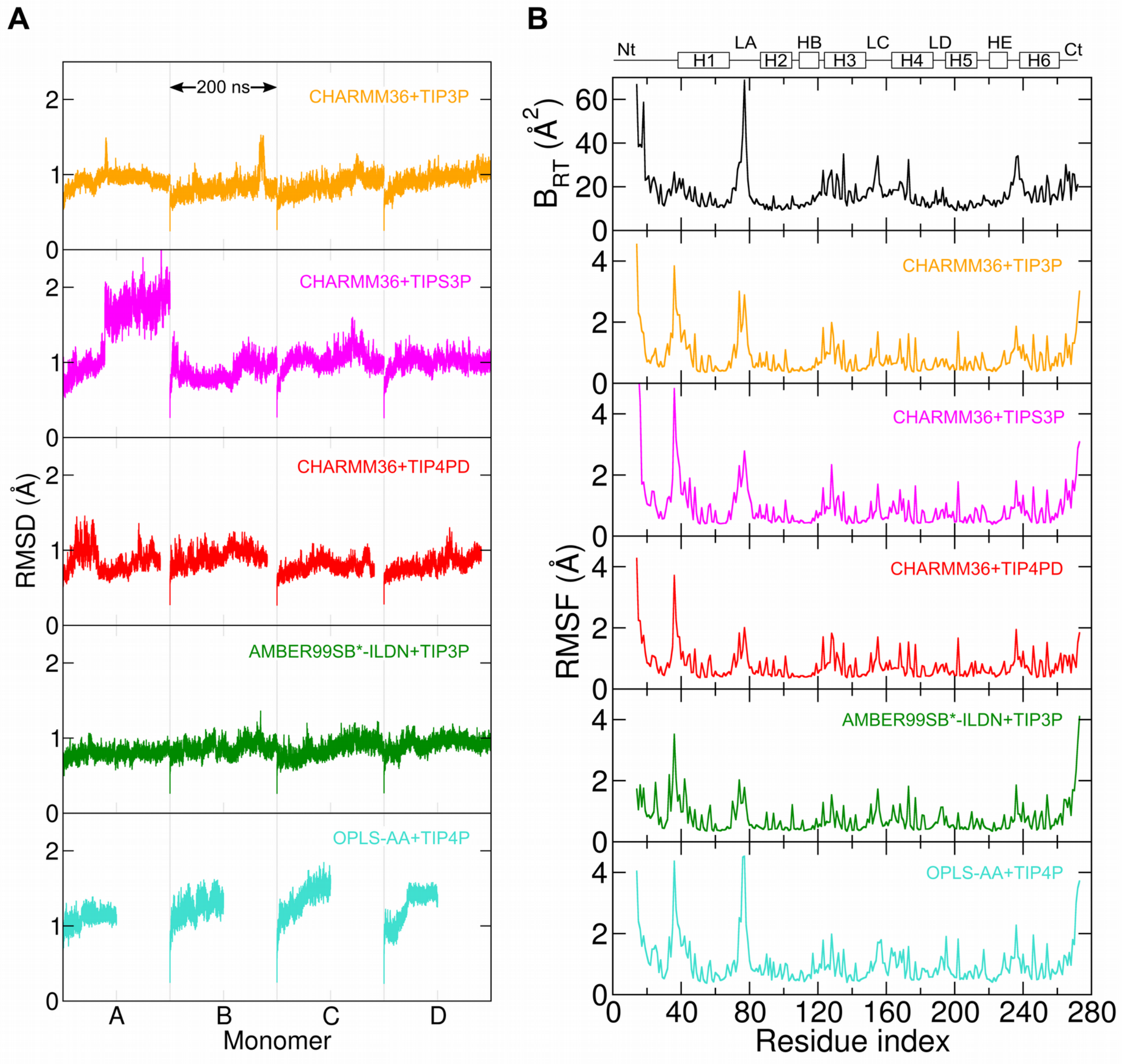

Fig. S1. A. Root mean square deviation (RMSD) as a function of the time recovered from equilibrium MD simulations of Aqy1 at room temperature. RMSD with respect to the initial conformation (taken from the room temperature, RT, structure). RMSD for the four monomers (A, B, C, D) is presented for the indicated force-fields. The black arrow indicates a simulation length of 200 ns. **B.** Comparison of the measured B-factors of the X-ray RT structure (B_{RT}) with the root mean square fluctuations (RMSF) recovered from simulations with the indicated force-fields, as a function of the residue index. Secondary-structure elements of the Aqy1 monomer are highlighted at the top of the figure.

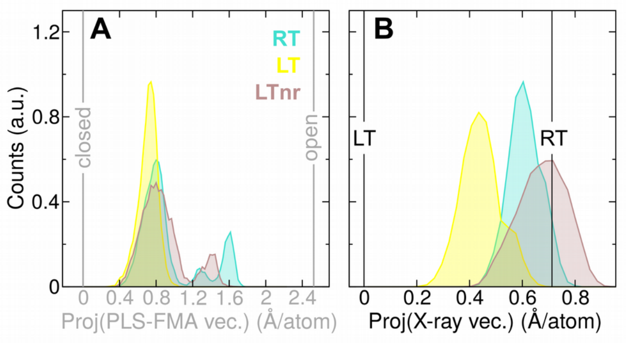

Fig. S2. Histograms of the projections of MD trajectories at room temperature along the PLS-FMA gating vector (A) and the vector connecting the X-ray structures (B), for three different starting conformations: taken from X-ray RT (cyan), refined LT (yellow), and non-refined LT, Lt_{nr} (brown) structures. The OPLS-AA+TIP4P force-field was used for this comparison.

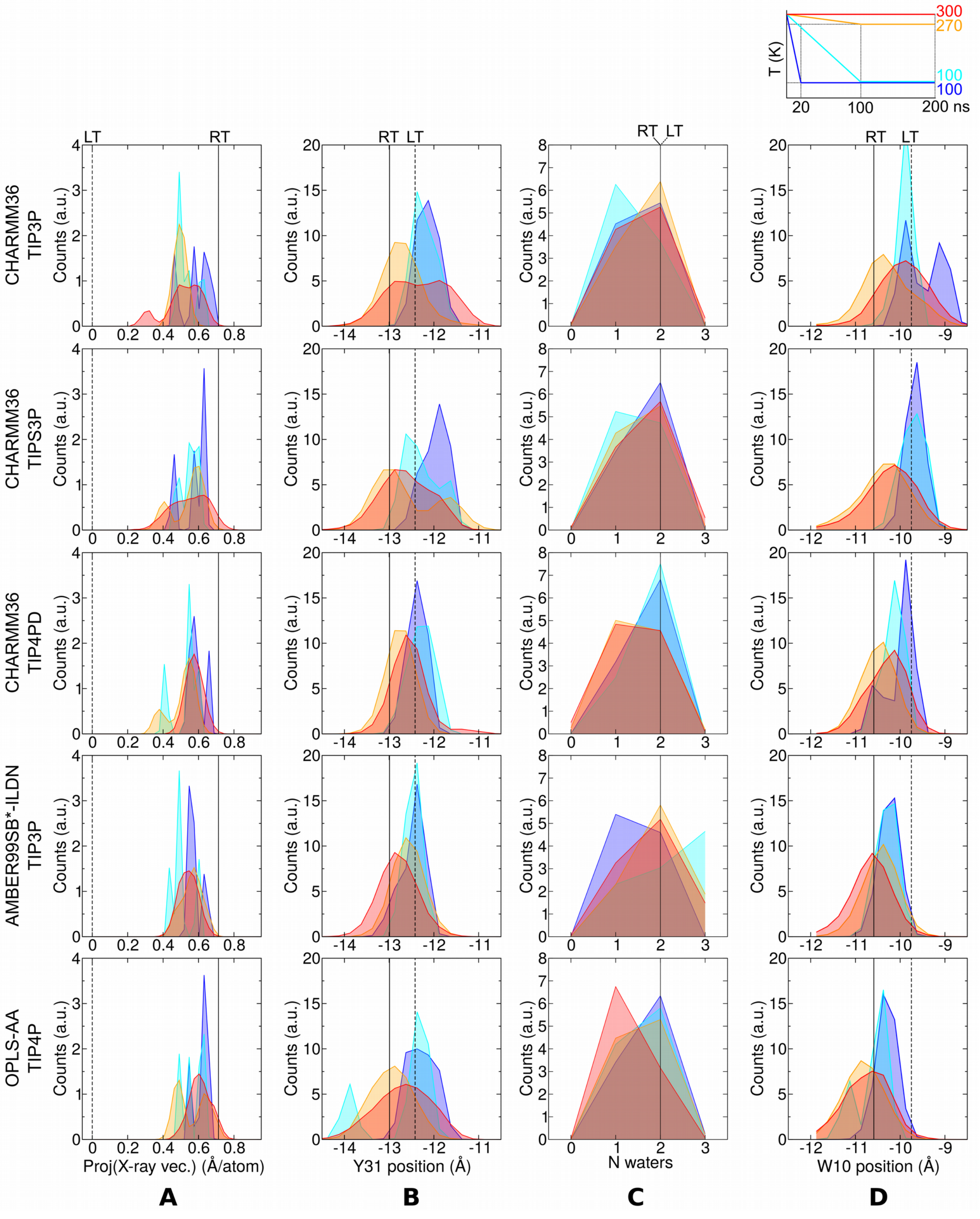

Fig. S3. Aqy1 freezing simulations for five different force-fields (different rows). Histograms of distinct quantities extracted from freezing MD simulations of Aqy1. **A.** Projection along the X-ray vector connecting the LT and RT structures. **B.** Position of the Tyr31 OH atom along the pore coordinate with respect to the NPA region. **C.** Number of waters coordinated by the asparagine residues Asn112 and Asn224 in the NPA region. **D.** Position of the water molecule at crystallographic position Wat10 with respect to the NPA region. Vertical lines represent the values observed in the X-ray structures. Initial temperature was 300 K. It was reduced to different temperatures as the time-trace sketched at the top. Histograms were computed during the last 80 ns of the simulations when the final temperature (different colors) was reached.

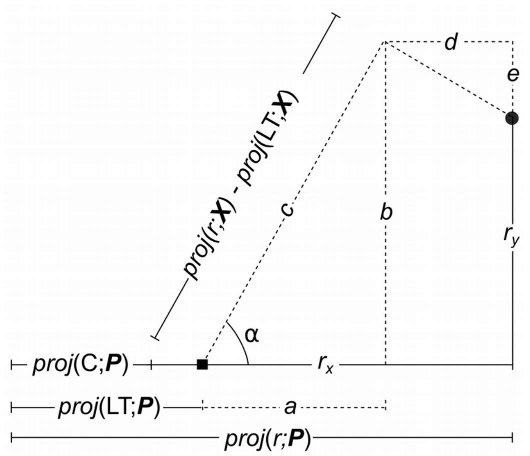

Fig. S4. Calculation of projections onto the two-dimensional space constituted by the PLS-FMA (***P***) and the X-ray (***X***) vectors. *P* goes along the horizontal axis. ***X*** forms an angle α with respect to ***P*** (parallel to segment *c*). The origin of the *X-axis* corresponds to the projection of the closed conformation C along ***P*** (*proj*(*C;**P***)). Projection of the low temperature conformation is taken as reference to determine the angle and the *y-axis* projection (indicated with the square). A point *r* with projections *proj*(*r*;***P***) and *proj*(*r*;***X***) onto the vectors ***P*** and ***X***, respectively, has coordinates (r_x, r_y) in the *XY*-plane. They can be deduced from the projections from trigonometry.

Supplementary text S1.

We used palmitoyloleoylphosphatidylcholine (POPC) lipids in the simulations with the AMBER99SB*-ILDN-TIP3P force-field, while we employed palmitoyloleoylphosphatidylethanolamine (POPE) lipids for all other simulations. However, we consider that the variation from PC to PE in the head-group of the surrounding lipids do not largely influence neither the overall structure of aquaporins nor affect their water-pore properties, as supported by high resolution cryo-EM data and MD simulations. Cryo-EM crystallography revealed the structure of the lipids surrounding aquaporin-0 [1; 2]. These studies determined that different types of lipids accommodated similarly around this aquaporin, maintaining the structure of the tetramer, and most importantly, of the water conduction pore almost unchanged. We complemented these findings by demonstrating that the acyl-chains rather than the head groups play a decisive role defining the lipid positions around aquaporin-0 [3] and that the protein mobility highly modulate the flexibility of the surrounding lipids but not vice versa [4]. The Multiscale simulations of Stansfeld *et al.* [5] revealed conserved patterns of lipid interactions with aquaporins, therefore implying that lipid-protein features observed for aquaporin-0 can be extrapolated to other aquaporins, including Aqy1. All this evidence strongly suggest, that the water-conduction pore of Aqy1, which is immersed in a rigid scaffold of transmembrane helices separated by about 1 nm from the surrounding lipids, is not largely affected by a moiety substitution in the head groups of the surrounding lipids.

REFERENCES

[1] Gonen, T.; Cheng, Y.; Sliz, P.; Hiroaki, Y.; Fujiyoshi, Y.; Harrison, S. C. and Walz, T. (2005). **Lipid-protein interactions in double-layered two-dimensional AQP0 crystals**, *Nature* 438 : 633-638.

[2] Hite, R. K.; Li, Z. and Walz, T. (2010). **Principles of membrane protein interactions with annular lipids deduced from aquaporin-0 2D crystals**, *The EMBO Journal* 29 : 1652-1658.

[3] Aponte-Santamaría, C.; Briones, R.; Schenk, A. D.; Walz, T. and de Groot, B. L. (2012). **Molecular driving forces defining lipid positions around aquaporin-0.**, *Proc. Natl. Acad. Sci. U. S. A.* 109 : 9887-9892.

[4] Briones, R.; Aponte-Santamaría, C. and de Groot, B. L. (2017). **Localization and Ordering of Lipids Around Aquaporin-0: Protein and Lipid Mobility Effects**, *Frontiers in Physiology* 8 : 124.

[5] Stansfeld, P.; Jefferys, E. and Sansom, M. (2013). **Multiscale Simulations Reveal Conserved Patterns of Lipid Interactions with Aquaporins**, *Structure* 21 : 810-819.
